# High-Resolution Lipidomics of the Early Life Stages of the Red Seaweed *Porphyra dioica*

**DOI:** 10.3390/molecules23010187

**Published:** 2018-01-17

**Authors:** Elisabete da Costa, Vitor Azevedo, Tânia Melo, Andreia M. Rego, Dmitry V. Evtuguin, Pedro Domingues, Ricardo Calado, Rui Pereira, Maria H. Abreu, Maria R. Domingues

**Affiliations:** 1 Centro de Espectrometria de Massa, Departamento de Química & QOPNA, Universidade de Aveiro, Campus Universitário de Santiago, 3810-193 Aveiro, Portugal; elisabetecosta@ua.pt (E.d.C.); vitorazevedo@ua.pt (V.A.); taniamelo@ua.pt (T.M.); p.domingues@ua.pt (P.D.); 2 ALGAplus—Produção e Comercialização de Algas e seus Derivados, Lda., 3830-196 Ílhavo, Portugal; amrego@algaplus.pt (A.M.R.); rgpereira@algaplus.pt (R.P.); htabreu@algaplus.pt (M.H.A.); 3 Departamento de Química & CICECO, Universidade de Aveiro, Campus Universitário de Santiago, 3810-193 Aveiro, Portugal; dmitrye@ua.pt; 4 Departamento de Biologia & CESAM & ECOMARE, Universidade de Aveiro, Campus Universitário de Santiago, 3810-193 Aveiro, Portugal; rjcalado@ua.pt

**Keywords:** *Porphyra dioica*, conchocelis, lipidome, glycolipids, phospholipids, betaine lipids, seaweeds, mass spectrometry, HILIC–ESI–MS

## Abstract

*Porphyra dioica* is a commercial seaweed consumed all over the world, mostly in the shape of nori sheets used for “sushi” preparation. It is a well-known part of the Asian diet with health benefits, which have been associated, among others, to the high levels of *n*-3 and *n-*6 fatty acids in this red alga. However, other highly valued lipids of *Porphyra* are polar lipids that remain largely undescribed and can have both nutritional value and bioactivity, thus could contribute to the valorization of this seaweed. In this context, the present work aims to identify the lipidome of two life cycle stages of the Atlantic species *Porphyra dioica*: the early life stage conchocelis produced in an indoor-nursery, and young blades produced outdoors using an integrated multitrophic aquaculture (IMTA) framework. Both the blades (gametophyte) and conchocelis (sporophyte) are commercialized in the food and cosmetics sectors. Liquid chromatography coupled to Q–Exactive high resolution-mass spectrometry (MS) platform was used to gain insight into the lipidome of these species. Our results allowed the identification of 110 and 100 lipid molecular species in the lipidome of the blade and conchocelis, respectively. These lipid molecular species were distributed as follows (blade/conchocelis): 14/15 glycolipids (GLs), 93/79 phospholipids (PLs), and 3/6 betaine lipids. Both life stages displayed a similar profile of GLs and comprised 20:4(*n*-6) and 20:5(*n*-3) fatty acids that contribute to *n*-3 and *n*-6 fatty acid pool recorded and rank among the molecular species with higher potential bioactivity. PLs’ profile was different between the two life stages surveyed, mainly due to the number and relative abundance of molecular species. This finding suggests that differences between both life stages were more likely related with shifts in the lipids of extraplastidial membranes rather than in plastidial membranes. PLs contained *n*-6 and *n*-3 precursors and in both life stages of *Porphyra dioica* the *n*-6/*n*-3 ratio recorded was less than 2, highlighting the potential benefits of using these life stages in human diet to prevent chronic diseases. Atherogenic and thrombogenic indexes of blades (0.85 and 0.49, respectively) and conchocelis (0.34 and 0.30, respectively) are much lower than those reported for other Rhodophyta, which highlights their potential application as food or as functional ingredients. Overall, MS-based platforms represent a powerful tool to characterize lipid metabolism and target lipids along different life stages of algal species displaying complex life cycles (such as *Porphyra dioica*), contributing to their biotechnological application.

## 1. Introduction

*Porphyra* spp. (Bangiales, Rhodophyta) is among the most important commercial seaweeds, being massively produced and traditionally consumed in Asia for nutrition and with recognized human health benefits [1,2]. In Western countries, the consumption of *Porphyra* (Nori) is increasing, mainly due to the “sushi” trend but also for being considered as a super food with health promotion benefits [2]. The increasing demand prompted by a higher consumption coupled with the need of high quality biomass, and locally sourced biomass, has been promoting the aquaculture of the genus *Porphyra* in the United States, South America and Europe. *Porphyra umbilicalis*, *Porphyra columbina* and *Porphyra dioica* are among the targeted species. *Porphyra dioica* (J. Brodie & L. M. Irvine, 1997-World Register of Marine Species) [3] is native to the Atlantic and its cultivation potential started to be explored in the late 90’s [4,5], being very well adapted to aquaculture [4]. To our knowledge, its commercial production is currently only done in a land-based integrated multitrophic aquaculture (IMTA) system in the west coast of Portugal, that has completed the *P. dioica* life cycle and selected varieties suitable for year-round organic production of conchocelis and blades [4,5]. 

**Figure 1:**
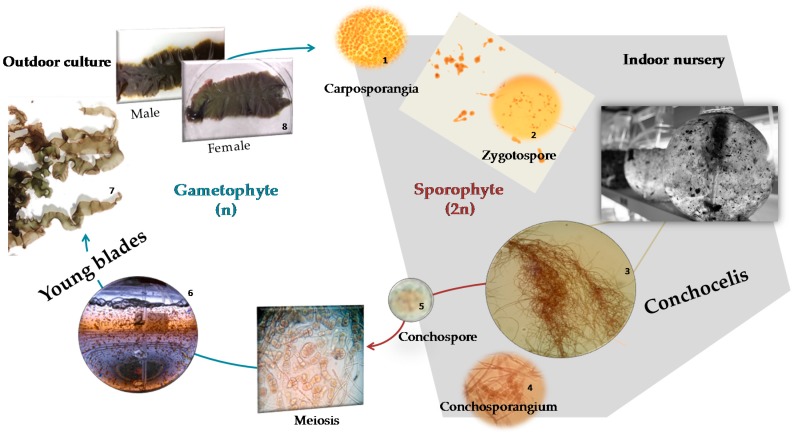
*Porphyra dioica* life cycle: the gametophytic blade phase reproduces sexually through fertilization of the carpogonium by the spermatium and subsequent formation of carpospores; the development of these spores gives rise to the filamentous sporophyte conchocelis phase, which produces conchospores that are release into the seawater and germinate to form new blades.

*Porphyra dioica* has a unique trimorphic life history, with two of those stages being analyzed in the present work: the blade and conchocelis phases (Figure 1) [6,7]. 

Blades are the gametophyte phase of *Porphyra* spp., being the most commercially relevant stage and used mainly for the food, feed, and other high value biotechnological applications [8,9]. *Porphyra* species have high nutritional value and are a valuable source of a plethora of metabolites such as peptides, sulfated galactans, and polysaccharides [10], or valuable lipids such as *n*-3 and *n*-6 polyunsaturated fatty acids (PUFAs) [11]. Lipids from seaweeds are majority composed by polar lipids that are a source of esterified fatty acids (FA) [12]. Some studies suggested that they have a vast array of biological activities [13,14,15]. Polar lipids from *Porphyra* sp. were reported to display anti-inflammatory properties by downregulation of LPS-induced pro-inflammatory responses in human macrophages through inhibition of IL-6 and IL-8 production [13,16] and antitumoral activities such as telomerase-inhibition effect [17]. However, there is still a gap on the knowledge of its lipidome and related health benefits. The early-stage filaments of *Porphyra*, conchocelis, are already used in cosmetics but not routinely consumed for food and are, at present, being surveyed by researchers, as it can be a potential source of PUFAs namely 20:5(*n*-3) FA [18,19]. After a hard process for obtaining clean and stable cultures, conchocelis can be grown by vegetative propagation under controlled conditions, with the advantages of exhibiting a fast growth and controlled production for standardized biomass [1,18,20]. In fact, and similarly to microalgae [8,21], conchocelis can be used to produce biomass enriched in target compounds such as lipids with nutritional and biological added-value and for distinct applications [2,10,17,22,23]. The conchocelis phase from Asian *Porphyra* species (now *Pyropia*) are typically cultivated on oyster shells [2]. However, in the case of *Porphyra dioica*, conchocelis are being produced indoors at specialized nurseries, while its potential for massive outdoor cultivation is currently being evaluated [5]. 

Lipids from seaweed are considered a source of valuable compounds for the food and pharma and have been bioprospected worldwide [24]. Glycolipids are associated with potential health benefits and that can be used as food and feed, as well as for a number of biotechnological applications [13,25,26]. Phospholipids have been referred as not only a superior nutritional source of *n*-3 PUFAs for functional food industries, but also for pharmaceutical applications due to the beneficial effects on the central nervous system and their antitumoral properties [27,28,29,30]. These molecules are also used as vehicles or carriers in dermatologic delivery systems and skin moisturizing products in the cosmetics industry [27]. 

The lack of knowledge on the lipidome of *Porphyra* species, at a molecular level, has encouraged us to identify and characterize the polar lipid profile of the conchocelis phase (indoor nursery) and young blades (outdoor cultures) of *Porphyra dioica* cultivated on a land-based integrated multitrophic aquaculture (IMTA) system. To achieve this characterization, we have used hydrophilic interaction chromatography coupled to Q–Exactive high resolution-mass spectrometry instrument (LC–MS) and gas chromatography coupled to mass spectrometry (GC–MS). These approaches allowed to unveil the composition of these distinct life stages of *Porphyra dioica* and contributed to a better understanding on the lipid dynamics along the life cycle of this seaweed. Moreover, this study provided valuable clues on the potential bioactive properties and nutritional value as source of *n-*3 and *n*-6 FA for the different life stages of this seaweed. Thus, overall, the results obtained foster the valorization of *Porphyra dioica* for its traditional use as food but also as a source of high value compounds for biotechnological applications.

## 2. Results and Discussion

The lipid extracts of the two life stages of *Porphyra dioica* analyzed in the present study accounted for about 8600 ± 120 mg kg^−1^ dry biomass of blade and 10,800 ± 90 mg kg^−1^ dry biomass of conchocelis. These results are in accordance with those previously reported for *Porphyra* spp. in other studies, which ranged between 2500–10,300 mg kg^−1^ of biomass [31,32].

### 2.1. Fatty Acids Profile from Porphyra Dioica

The fatty acid profile of the lipid extracts was obtained for the two phases. Fifteen different fatty acids were identified in *P. dioica* blade and conchocelis stages (Table 1). Among these were 14:0 (myristic acid), 15:0 (pentadecanoic acid), 16:0 (palmitic acid), 16:1 (palmitoleic acid), 18:0 (stearic acid), 18:1(*n*-9) (oleic acid), 18:1(*n*-7) (vaccenic acid), 18:2(*n*-6) (linoleic acid), 20:1(*n*-9) (eicosenoic acid), 20:2(*n*-6) (eicosadienoic acid), 20:3(*n*-6) (dihomo-gamma-linolenic acid), 20:4(*n*-6) (arachidonic acid, AA), 20:5(*n*-3) (eicosapentaenoic acid, EPA), 22:1(*n*-9) (erucic acid). Both life stages displayed the same composition in terms of FAs, but differed in the content of some of the fatty acids identified. The blade phase was characterized by higher amounts of SFA (942.3 mg kg^−1^ dry biomass, representing 46.1% of total fatty acids) mainly due to the high content of 16:0 (882.1 ± 12.4 mg kg^−1^ dry biomass, representing 43.2% of total fatty acids) and PUFA (775.9 mg kg^−1^ dry biomass, representing 38.0% of total fatty acids) with high amount of eicosapentaenoic acid 20:5(*n*-3) (518.3 ± 14.2 mg kg^−1^ dry biomass, representing 25.4% of total fatty acids). In comparison, conchocelis contained a higher amount of MUFA (948.3 mg kg^−1^ dry biomass, representing 26.5% of total fatty acids) and PUFA (1729 mg kg^−1^ dry biomass, representing 48.3% of total fatty acids), and was richer in AA 20:4(*n*-6) (751.0 ± 87.3 mg kg^−1^ dry biomass, representing 21.0% of total fatty acids), but also contained a high amount of 20:5(*n*-3) (579.5 ± 96.4 mg kg^−1^ dry biomass, representing 16.2% of total fatty acids). Conchocelis of *Porphyra dioica* contained alpha-linolenic acid 18:3(*n*-3) in low amounts, an important precursor of 20:5(*n*-3).

Comparing with available literature, *Porphyra* species (adult stages) typically display high concentrations of 20:5(*n*-3), with 20:4(*n*-6) being the second most abundant PUFA [32,33,34]. The percentage of 20:5(*n*-3) commonly ranges from 20 up to 50% of the total pool of FA [16,32,35]. This feature highlights the potential of *Porphyra dioica* as a commercial supply of *n*-3 EPA, assuming that sufficient biomass can be provided sustainably, such as in the case of cultivation in aquaculture systems [33,36,37]. 

The conchocelis stage displayed a higher content of 20:4(*n*-6) rather than 20:5(*n*-3), which is in agreement with the literature [18,20,35]. Both FA are within ranges previously reported: 23% up to 45% of 20:4(*n*-6) FA and 5% up to 26% of 20:5(*n*-5) [35]. Highest PUFA and MUFA, and lowest SFA contents were found in conchocelis phase, but the relative percentage of 20:5(*n*-3) of blade stage was higher than in conchocelis. It must be highlighted that *n*-3 FA have important roles on regulatory pathways controlling inflammatory responses [38,39]. Many studies reported that supplementation with EPA contribute to reduce the risk of acute inflammatory response [40,41]. General benefit to human health through anti-inflammatory actions can be achieved by reinforcing diet with EPA of somewhere between 1.35 and 2.70 g EPA per day [42].

The *n*-6/*n*-3 PUFA ratio determined in both life stages was 0.50 for blades and 1.90 for conchocelis. Overall, the *n*-6/*n*-3 ratio was less than 2 in both stages, and below the upper limit reported for *Porphyra* spp. (1.2 up to 9) or other seaweeds [18,32]. This ratio in *Porphyra dioica* is within the range recommend as beneficial (<4) to reduce chronic diseases [11,43,44,45]. This feature enhances its valorization to be used in human diet, as well as other potential application [18], since lipids provided by diet are recognized to promote health and complement therapies [11,40,42,46]. Atherogenic index and thrombogenic index (IA and IT) allow estimating the effects of dietary FA on human health and on coronary diseases, such in atheroma and/or thrombus formation [46]. The ratio between saturated thrombogenic FA and the sum of anti-thrombogenic MUFA and PUFA were calculated for the two *Porphyra* life stages (Table 1). Calculated IA and IT were higher in the case of blade stage (0.85 and 0.49 respectively) than in the case of conchocelis (0.34 and 0.30 respectively), but lower than reported for some Rhodophyta, Ochrophyta and Chlorophyta seaweeds [46,47]. Calculated IT values were similar to those obtained for other foodstuff considered beneficial for the prevention of chronic diseases, namely olive oil (0.32), chicken (0.95) and milk-based products (2.1).

Altogether, based on the FA profile, both *Porphyra dioica* blade and conchocelis are valuable sources of high add value lipids that can be used for food, as a food additive, in nutraceuticals or for inclusion in the formulation of highly unsaturated low-fat diets. Overall, they can help to prevent diseases by improving the thrombogenic and atherogenic potential and thus allow the development of healthier lipid formulations. 

### 2.2. Polar Lipids from Porphyra dioica

Lipid extracts from of *Porphyra dioica* blade and conchocelis stages were analyzed by lipidomic mass spectrometry coupled to liquid chromatograph approach. The interpretation of MS data & mass accuracy, the MS/MS spectra and analysis of typical fragmentation pattern provided the detailed structural information to identify the different lipid classes at molecular level in each life cycle stage. The analysis of LC–MS data allowed the identification of glycolipids (GLs), phospholipids (PLs), and betaine lipids *in Porphyra dioica* lipid extracts, as will be detailed.

#### 2.2.1. Glycolipids—GLs

**Figure 2:**
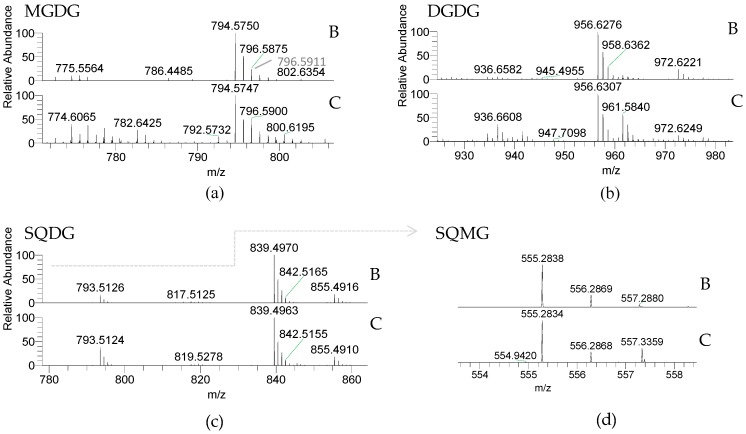
LC–MS of glycolipids from *Porphyra dioica* blade (B) and conchocelis (C) detected using HILIC–MS: (**a**) Monogalactosyl diacylglycerol (MGDG) and (**b**) Digalactosyl diacylglycerol (DGDG) molecular species were identified as [M + NH_4_]^+^ ions; (**c**) Sulfoquinovosyl monoacylglycerol (SQDG) and (**d**) Sulfoquinovosyl monoacylglycerol (SQMG) were identified as [M − H]^−^ ions.

Glycolipids (GLs) were observed in both *Porphyra dioica* life stages and included the neutral galactolipids 1,2-diacyl-3-*O*-(β-d-galactopyranosyl)-*sn*-glycerol, abbreviated monogalactosyl diacyl-glycerol (MGDG) and 1,2-diacyl-3-*O*-(α-d-galactopyranosyl-(1→6)-*O*-β-d-galactopyranosyl)-*sn*-glycerol, or digalactosyl diacylglycerol (DGDG), and the acidic sulfolipids 1,2-diacyl-3-*O*-(6-sulfo-6-deoxy-α-d-glucosyl)-*sn*-glycerol herein called sulfoquinovosyl diacylglycerol (SQDG) and the lyso-SQDG, the sulfoquinovosyl monoacylglycerol (SQMG) (Table 2, Figure 2). 

GLs are important membrane lipids located in photosynthetic membranes (thylakoid and chloroplast membranes) [48,49]. MGDG and DGDG were identified by LC–MS as [M + NH_4_]^+^ ions and the MS/MS spectra showed the typical fragmentation pathways of these classes of neutral glycolipids, namely product ions resulting from the loss of NH_3_, loss of hexose residue (−162 Da, −Hex_res_), and loss of hexose moiety (−180 Da, −Hex) [50,51,52]. Product ions formed by combined loss of one FA and the hexose moiety yield a typical acylium ions plus 74 [RCO + 74]^+^ that allows to identify the FA composition. MGDG included five and six molecular species and DGDG contained four molecular species assigning blade and conchocelis lipidome, respectively. Both life stages contained the abundant MGDG (20:5/16:0) at *m*/*z* 794.6. The MGDG (20:4/16:0) and MGDG (18:1/16:0) (*m*/*z* 796.6 and *m*/*z* 774.6, respectively) were also abundant in conchocelis extracts and a source of eicosapolyenoic acid 20:4(*n*-6). MGDG (18:1/18:0) at *m*/*z* 802.6 was not observed in blade lipidome. The most abundant DGDG in both stages was DGDG (20:5/16:0) at *m*/*z* 956.6. In the case of conchocelis the second most abundant molecular species was the DGDG (18:1/16:0) at *m/z* 936.7, matching with results observed in the MGDG class. The other molecular species included 18:2 FA, a precursor of the omega-pathway [53,54]. 

The anionic sulfolipids SQDG and SQMG were identified in the LC–MS spectra of both stages as [M − H]^−^ ions. Structural characterization of molecular species of these acidic glycolipids was achieved by MS/MS interpretation. Their MS/MS spectra showed the typical product ion at *m*/*z* 225.007, attributed to the sulfoquinovosyl anion, and also showing the typical product ions due to the loss of fatty acyl chains both as acid (−RCOOH) and ketene (−R=C=O) [50,51,52]. The product ions attributed to carboxylate anions (RCOO^−^) are also present in the MS/MS spectra of the molecular species of SQDG and SQMG allowing to corroborate the identification of the fatty acyl composition of each molecular species. Overall, four SQDGs were identified in both stages. The SQDG (20:5/16:0) at *m*/*z* 839.5 was the most abundant. Other molecular species combining 16:0 plus 18:2, 20:4 and 20:5 FA were observed. Only one SQMG (16:0), a lyso-SQDG, was identified in the lipidome of blades and conchocelis (*m*/*z* 555.3). Sulfolipids have a functional role in the “plastid membrane mosaic” that relies particularly in signaling and coordinating between chloroplast lipids and cytosolic partners [55].

The GLs profile is similar, showing the same glycolipid molecular species between the two life stages of *Porphyra dioica* life cycle, except MGDG (36:1) only detected in conchocelis. Since GLs are found in chloroplasts, during the early development stages of this seaweed, the formation of chloroplasts, structural rearrangement and development of plastid structure occurs but seems to preserve the GLs profile. Both life stages contained a lower number of SQDGs in comparison with other Rhodophyta already studied by using the same lipidomic analytical approach, such as *Chondrus crispus* [50] or *Gracilaria* sp. [51]. This could be a putative taxonomic biomarker. Also by using distinct MS- [51,52] and NMR-approaches [15], it was reported that GLs from Rhodophyta (e.g., *Chondrus crispus*, *Gracilaria* species, *Palmaria palmata*, *Porphyra* spp., among others) contained a range of molecular species combining the eicosapolyenoic acids 20:4(*n*-6) and 20:5(*n*-3). This was observed herein since GLs of *Porphyra dioica* contribute to the pool of balance 20:4(*n-*6)/20:5(*n-*3). These features endorse the potential applications of GLs from this seaweed. In fact, GLs bearing *n*-3 PUFA were reported with antibacterial, antitumoral, and antiviral activities, enhancing the pharmacological potential of these compounds [13,41]. Molecular species such as MGDG (20:4/16:0), MGDG (20:5/16:0), and SQDG (20:5/14:0) are recognized by their anti-inflammatory and antitumoral activity [15,51,56]. Altogether these structures provide important clues related to the potential bioactive properties of both lipid extracts and GLs from *Porphyra dioica* and also as an important source of *n*-3 and *n*-6 FA, fostering the valorization of these seaweeds.

#### 2.2.2. Phospholipids (PLs)

**Figure 3:**
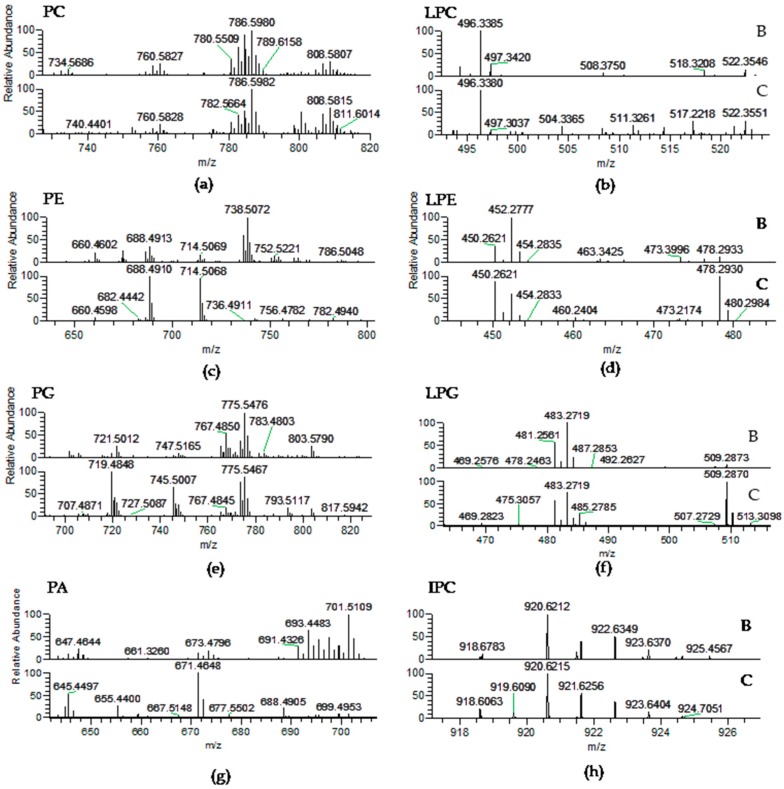
LC–MS spectra of phospholipid classes: (**a**) Phosphatidylcholine (PC); and (**b**) Lyso-PC (LPC) as [M + H]^+^ ions; (**c**) Phosphatidylethanolamine (PE); (**d**) Lyso-PE (LPE); (**e**) Phosphatidylglycerol (PG); (**f**) Lyso-PG (LPG); (**g**) Phosphatidic acid (PA); and (**h**) Inositolphosphoceramide (IPC) detected as [M − H]^−^ ions in *P. dioica* blade (B) and conchocelis (C).

The PL classes identified in *Porphyra dioica* were phosphatidic acid (PA), phosphatidylglycerol (PG), lyso-PG (LPG), phosphatidylcholine (PC), lyso-PC (LPC), phosphatidylethanolamine (PE), lyso-PE (LPE), phosphatidylinositol (PI), and inositephosphoceramide lipids (IPC) (Table 3, Figure 3). PLs are major components of cell membranes [57]. They play important roles in cell membranes to maintain the integrity and act as structural entities confining subcellular components [48]. Among PLs, PC and LPC were identified in the LC–MS spectra as [M + H]^+^ ions (Table 3, Figure 3a) and the typical fragmentation pattern was observed in the corresponding MS/MS spectra, namely the product ion at *m*/*z* 184.074 attributed to the phosphocholine headgroup [50,51,52]. Product ions formed by the cleavage of the ester bonds linking alkyl chains to the glycerol moiety were also observed and allowed to confirm the FA composition. In *Porphyra dioica*, twenty-seven PCs were identified in the blade stage and seventeen PCs in conchocelis by LC–MS. The most abundant PC was common to both stages and attributed to PC (18:1/18:1) at *m*/*z* 786.6, with minor contribution of PC (18:0/18:2). The second most abundant PC species in the lipidome of blades was PC (16:0/20:3) at *m*/*z* 784.6, with minor contribution of PC (18:1/18:2), while in the lipidome of conchocelis the second most representative was PC (18:1/20:4) at *m*/*z* 808.6. Thus, the blade stage can be distinguishable from conchocelis by the number of PC molecular species identified and profile (Figure 3a). Particularly, the blade stage included molecular species combining fatty acyl 14:0/18:1, 16:0/16:1, 16:0/18:0, 18:0/18:1, 18:0/20:3, and 18:3/20:4 plus 18:2/20:5 that were absent on the lipidome of conchocelis, contributing to a slightly higher number of PCs on blades. Some of these molecular species present in blades included the important 18:3 and 20:5(*n*-3) FA and 16:0 FA related to chloroplastic biosynthesis of lipids. Moreover, both stages have molecular species with the 20:3 FA that along with 18:3 FA are precursor of the *n*-3 and *n*-6 pathways. Both stages have also lyso-PC, similarly as described for *Porphyra haitanensis* [58], as well as other Rhodophyta [50,51]. Five LPC molecular species were identified in blade and two LPC assigned conchocelis with most abundant LPC (16:0), observed at *m*/*z* 496.3 (Table 3, Figure 3b).

The classes PE and lyso-PE were identified in the MS spectra as [M − H]^−^ ions (Table 3, Figure 3c) and their fragmentation pattern in the MS/MS spectra included product ions attributed to glycerophosphoethanolamine anion at *m*/*z* 196.037 and the phosphoethanolamine anion at *m*/*z* 140.010 plus carboxylate anions of fatty acyl chains (RCOO^−^) [51]. Eleven PEs were identified in the lipidome of blades while thirteen PEs were found in conchocelis. Herein, it was observed a large differentiation between the profile of molecular species of PE between stages. The most abundant species found in the blade was PE (16:0/20:4) at *m*/*z* 738.5 that was not assigned in the profile of conchocelis. Nevertheless, PE (16:0/16:1) and PE (16:1/18:1), respectively at *m*/*z* 688.5 and 714.5, were the most abundant PE in conchocelis stage. In common, both stages included the molecular species with acyl composition C_14–16_/C_14–20_, while their differentiation was based on the higher number of PEs combining C_18_/C_18_ moieties, absent in the lipidome of the blade stage (e.g., PE (18:0/18:1)). Meanwhile, PE (20:4/20:4) was only identified in the lipidome of the blade. Three lyso-PEs were identified on both stages, however LPE 16:0 at *m*/*z* 452.3 was the most abundant molecular species on the blade, while LPE 16:1 and LPE 18:1 (*m*/*z* 450.3 and 478.3) were higher on conchocelis (Table 3, Figure 3d). The non-photosynthetic PE class can be a precursor of PC and also play important roles in the maintenance of shape and curvature of cell membranes and are important to folding of some membrane proteins as well as to establish protein-lipid conjugates [48,59,60]. Differences observed in PE profile suggest a key relationship with different properties of cell membrane and organelles observed in early-stages vs. more developed stages. In both stages studied in the present work, a higher number of PE species were identified when compared with data previously reported for the lipidome of *Gracilaria* sp. (by using similar analytical approaches) [51]. Further studies are needed to better understand if this dissimilarity could be due to taxonomic differentiation.

Phosphatidylglycerol (PG) and lyso-PG (LPG) were identified as [M − H]^−^ ions. The MS/MS spectra showed the typical neutral loss of 74 Da and the formation of the product ions such as glycerol phosphate anion at *m*/*z* 171.006 together with glycerophosphate glycerol anion minus H_2_O at *m*/*z* 227.032 confirm the class. Product ions attributed to carboxylate anions (RCOO^−^) of FA were also found in the MS/MS spectra [50,51,52]. Twenty PGs were identified in the blade, while twenty-seven PGs were assigned in the conchocelis lipidome (Table 3, Figure 3e). In the lipidome of conchocelis, the most abundant PG was at *m*/*z* 719.5 corresponding to PG (16:1/16:0) with minor contribution of PG (14:0/18:1), followed by PG (18:0/18:1) at *m*/*z* 775.5, with minor contribution of PG (16:0/20:1). It is worth highlighting that PG (18:0/18:1) at *m*/*z* 775.5, was the most abundant in the blade stage and PG (16:0/20:5) at *m*/*z* 767.5 with minor contribution of PG (16:1/20:4) was also abundant in the PG profile of this life stage of *Porphyra dioica*. Overall, both stages contained PG molecular species that combine C_16_ with C_14–22_ and C_18_ with C_14–22_ of SFA, MUFA and PUFA, but the relative abundances of the molecular species change with the life stage. PG is involved in the formation of chloroplast’s membrane and are directly related to the biosynthesis of SQDG [12,61]. The profile of PG from the conchocelis stage was differentiated by the presence of more four molecular species (Table 3). Three lyso-PG are present in both stages, with the most abundant in blade corresponding to LPG (16:0), while both LPG (16:0) and LPG (18:1) are abundant in the lipidome of conchocelis (Table 3, Figure 3f). The PA class was identified in negative-mode as [M − H]^−^ ions and confirmed by MS/MS spectra that showed the typical product ion of glycerophosphate (*m*/*z* 152.995) and the product ions attributed to carboxylate anions (RCOO^−^) [50,51,52]. Seventeen molecular species were identified in the lipidome of the blade, while only six were detected in conchocelis (Table 3, Figure 3g). The most abundant PA observed in the blade was PA (16:0/20:1) at *m*/*z* 701.5, while the second most abundant was observed at *m*/*z* 693.4 and corresponded to PA (16:0/20:5). The predominant PA in the conchocelis lipidome was detected at *m*/*z* 671.5 and assigned to PA (16:0/18:2) and at *m*/*z* 645.4 corresponding to PA (16:0/16:1) plus PA (14:0/18:1). The profile of molecular species of PA in the lipidome of both stages included distinct combinations between 16:0 and 16- to 20-carbon chains FA. The number of molecular species is significantly higher in the blade stage and the profile of molecular species is different between both stages. The PA class is a vital cell biosynthetic precursor for all chloroplastic and extra-plastidial glycerolipids and a key signaling lipid that regulates numerous reactions involved in development and in response to environmental stresses [48,62,63]. In fact, different roles of PA in the multiple cellular functions of macroalgae membranes are points that will be interesting to full explore.

Phosphatidylinositol (PI) molecular species were identified in negative-mode as [M − H]^−^ ions and structural characterization was confirmed by the analysis of the MS/MS spectra that showed the typical product ions at *m*/*z* 223.001 and *m*/*z* 241.012 attributed to the inositol headgroup and also the product ions attributed to carboxylate anions (RCOO^−^) [50,51,52]. Three PIs, were identified in the blade and five PIs were recorded in conchocelis lipidome (Table 3). Both stages included PI (16:0/18:2) with contribution of PI (16:1/18:1) at *m*/*z* 833.5 and PI (16:0/18:1) at *m*/*z* 835.5. Other molecular species of PI that fingerprinted the lipidome of conchocelis included *n*-3 FA: PI (16:0/18:3) and PI (18:0/20:5). PIs are usually low abundant phospholipids in biological matrices [64] although they play important regulatory roles in metabolism [65]. PIs are involved in various cellular processes, such as in the control of membrane trafficking, cytoskeletal remodeling, ion transport and signal transduction [65,66,67]. These polar lipids have essential roles as signaling molecules, in the maintenance of cell polarity, and during cell-cycle phases [20]. These molecular species were found in the phospholipidome of other Rhodophyta [48,51,61]. Phosphoinositides such as inositephosphoceramides (IPC) were identified in *Porphyra dioica* as [M − H]^−^ ions, in both life stages studied. LC–MS/MS spectra of the [M − H]^−^ ions of IPCs showed the typical fragmentation pattern showing the losses of 162 Da and 180 Da, due to elimination of inositol, the product ion resulting from the loss of fatty acyl chains, and the product ion at *m*/*z* 259.022 that corresponded to the inositol monophosphate anion [50,51]. Four molecular species were detected in the blade and three in conchocelis lipidomes (Table 3, Figure 3h). The most abundant IPC in blades was observed at *m*/*z* 920.6 (IPC (t42:2-OH)), with the molecular species IPC (d18:1/26:0) being absent in the lipidome of conchocelis (Table 3, Figure 3h). Other molecular species included phytosphingosine t18:0 and C_24_-OH. Interestingly, IPCs were reported for the first time for *Gracilaria verrucosa* [68], and latter for *Chondrus crispus* and *Gracilaria* sp. [50,51] and are considered a putative biomarker of Rhodophyta because IPC was not identified for the other phyla.

A plethora of molecular species from PLs distinct classes were identified for *Porphyra dioica*. PLs such as PC, LPC and PA contained a huge range of molecular species in the blade, while PG and PE contained a higher number of molecular species in the lipidome of conchocelis. Both stages included large numbers of polar lipids combining 14-, 16-, 18-, and 20-carbon chain FA. Overall, dissimilarities of the phospholipidome were observed between the two life stages of *Porphyra dioica* due to the number of molecular species and their abundance, which suggested that some shift in the lipid metabolism across the life cycle of this seaweed. It is well established that PC, PE, PG, and PI are related to the formation of new cells and organelles, supporting the variation on the profiles of molecular species recorded between the two life stages surveyed [69]. The profile of PLs is regulated in order to maintain membranes structure and function, in spite of growth and environment changes [69,70,71]. Moreover, higher number of lyso-phospholipids from PC, PG and PE were found in the lipidomes of this seaweed than lyso-glycolipids, and a higher number of molecular species of lyso-PC in blade suggest that phospholipases were activated to a greater extent than galactolipases [58].

Comparing with the lipidome of other red seaweeds, which have already been analyzed by LC–MS [50,51], a higher number of molecular species of PE were identified in *Porphyra dioica*. This finding, along with the presence of LPE, suggest that these lipid molecular species can be putative taxonomic biomarkers to identify *Porphyra dioica* within the Rhodophyta. These results are worth to be further explored for traceability of cultured seaweeds.

At present, PLs applications are entering a new area in the food industry, mainly as an ingredient for food fortification. PLs may serve important functions within the functional food segment due to their: (a) emulsifying properties; (b) ability to supplement *n*-3 FAs such as 20:5(*n*-3); (c) enhanced bioavailability of PUFA; and (d) intrinsic beneficial nutritional effects [30]. PLs can be used as natural emulsifiers that facilitate the digestion and absorption of fatty acids, cholesterol and other lipophilic nutrients [21]. Furthermore, marine PLs emulsions can be used as carriers of *n*-3 PUFA and incorporated easily into aqueous and emulsified foods. Besides their nutraceutical relevance, there is also a growing interest in the use of PLs from seaweeds in the cosmetic and pharmaceutical industries, not only as emulsifiers but also due to their health benefits [14,16,52]. At present, *n*-6 and *n*-3 long chain PUFA incorporated into PL classes are already known to play important biological functions, namely alleviate senescence, to be beneficial for cognitive functions or prevent inflammatory diseases [27]. Anti-inflammatory effect of PLs rich-extracts were attributed to *n*-3 and *n*-6 PUFA and to some molecular species of polar lipid bearing these FAs, namely PG (20:5/16:0), PG (20:5/16:1) and PC (20:5/20:5) [15]. Choline from the headgroup of PC seems to play important roles in the stimulation of the production of acetylcholine with beneficial impact on the central nervous system [27]. Thus, seaweeds or isolated PLs used as functional food or ingredients, afford diverse opportunities to improve health.

#### 2.2.3. Betaine Lipids

**Figure 4:**
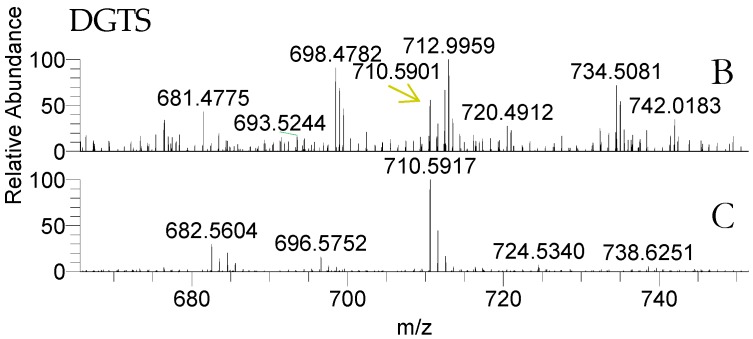
LC–MS of diacyglyceryl-*N*,*N*,*N*-trimethyl homoserine (DGTS) as [M + H]^+^ ions in the LC–MS spectra of blade (B) and conchocelis (C).

Both life stages of *Porphyra dioica* contained betaine lipids diacyglyceryl-*N*,*N*,*N*-trimethyl homoserine (DGTS) (Table 4, Figure 4). The analysis of total lipid extract provided the identification of some DGTS, identified as [M + H]^+^ ions. Structural characterization was achieved by analysis of the MS/MS spectra that showed the characteristic product ion at *m*/*z* 236.150 that results from the combined loss of the two fatty acyl chains as ketenes plus product ions attributed to the loss of a single fatty acyl chains as ketene [50,51,52]. A total of six molecular species were identified in conchocelis stage and only three were identified in blade samples (Figure 4). The most abundant molecular species in conchocelis corresponded to DGTS (16:0/16:1) at *m*/*z* 710.6, with minor contributions of DGTS (14:0/18:1) (Figure 4 and Table 4). These molecular species were also recorded in the blade lipidome, that also include other DGTS molecular species combining 14:0, 16:0, 16:1 and 18:2 FA, but not eicosapolyenoic 20:4 and 20:5 FA.

Betaines were recently identified by LC–MS in the lipidome of the Rhodophyta *Chondrus crispus* and *Gracilaria* sp. [50,51]. Kunzler, by using TLC-based approaches, also detected DGTS in small amounts in orders Gigartinales, Rhodymeniales and Ceramiales, but not in order Bangiales [72], the order of *Porphyra dioica*. This finding suggests that, at least in some red macroalgae species, the biosynthetic pathway for betaine lipids is indeed effective but likely not contributing substantially to polar lipids as in Chlorophyta [73,74,75]. Betaine lipids are naturally occurring lipids not found in higher plants, but are quite widely distributed in algae [73,75]. They are components of extraplastidial membranes and of the outer membrane of chloroplasts [73], are involved in the transport of fatty acids from the cytoplasm to the chloroplast, and may contribute as a marker of environmental and nutrition depleted conditions [75,76]. They can replace PC phospholipids under phosphorous limitation in membranes and in cellular lipid metabolism, since both have the same positive charge choline in their polar head groups [72,75]. These are novel-lipids that are yet to be well explored in seaweeds. In fact, several gaps of knowledge remain to be addressed in the field of structural characterization, metabolic pathways, bioactivity and, consequently, the potential for further applications in a biotechnological perspective is still to be unraveled. The amphiphilic character of betaines is similar to that of PC, thus suggesting that some relation may exist in terms of biological role and can be important for bioprospection and applications. However, this is an unexplored field that need to be addressed in future studies.

**Figure 5:**
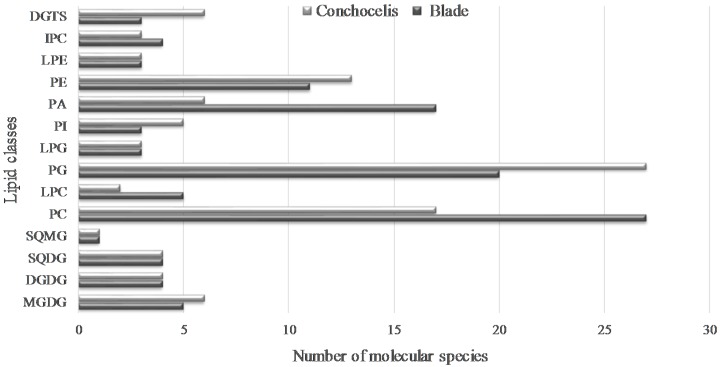
Number of individual lipids identified in the lipidome of blades and conchocelis of *Porphyra dioica*. Monogalactosyl diacylglycerol (MGDG), digalactosyl diacylglycerol (DGDG), sulfoquinovosyl monoacylglycerol (SQDG), sulfoquinovosyl monoacylglycerol (SQMG), phosphatidylcholine (PC), lyso-PC (LPC), phosphatidylglycerol (PG), lyso-PG (LPG), phosphatidylinositol (PI), phosphatidic acid (PA), phosphatidylethanolamine (PE), lyso-PE (LPE), inositephosphoceramide (IPC) and diacyglyceryl-*N*,*N*,*N*-trimethyl homoserine (DGTS).

Overall, 110 and 100 lipid molecular species fingerprint the lipidome of blade and conchocelis, respectively, of *Porphyra dioica* distributed over fourteen/fifteen glycolipids (GLs), ninety-three/seventy-nine phospholipids (PLs), and three/six betaine lipids (Figure 5). 

In different life stages, changes in lipid profile during the life history of *Porphyra* was mainly related to polar lipids from extraplastidial membrane: the number of polar lipids from PLs and betaine lipids were the ones that displayed most pronounced changes. Our study showed that the two life stages surveyed display dissimilar PLs profile mainly due to the high number of molecular species in PC, LPC, and PA in the blade and the higher number of PG and PE species in conchocelis. Betaine lipids from conchocelis contained high number of molecular species than blade. These findings confirm that, from a lipidomic point of view, the valorization of the conchocelis of *Porphyra dioica* may be relevant for culturing this species under controlled conditions. Indeed, conchocelis yields a number of lipid molecular species that complement the pool of lipids available solely through the processing of the blades of this red seaweed. Thus, the biomass of conchocelis that was once solely destined to secure the production of *Porphyra dioica* blades may also be channeled to other value chains that may significantly enhance the market value of this red seaweed.

## 3. Experimental Section

### 3.1. Materials

HPLC grade chloroform and methanol (MeOH) were purchased from Fisher Scientific Ltd. (Loughborough, UK). All other reagents were purchased from major commercial sources. Milli-Q water (Synergy^®^, Millipore Corporation, Billerica, MA, USA) was used. Methyl ester of fatty acids mixture was 37 Component FAME Mix from Supelco (Sigma Aldrich, St. Louis, MO, USA) and methyl heptadecanoate ≥99% was from Sigma (St. Louis, MO, USA). All standards used had a purity of ≥99% and were used without further purification.

### 3.2. Biomass

Seaweeds were provided by the SME ALGAplus Ltd. (production site in Ria de Aveiro, mainland Portugal, 40°36′43″ N, 8°40′43″ W). The gametophyte stage of *Porphyra dioica* (blade) were cultivated on IMTA tanks (1000 L) at ALGAplus under stable conditions of nutrients flow, pH (8.0–8.2), salinity (22.1 ± 1.46), and temperature (13.8 ± 0.93 °C). Seaweeds were cleaned in filtered seawater and dried at low temperature 25 °C) in an air tunnel, until reaching 10–12% total moisture. Dried material was milled (into 0.5–1 mm flakes). Samples of the seaweed were representative of the total biomass in production. The conchocelis phase of *P. dioica* was cultivated in the indoor nursery, in vegetative mode at 15 °C, an irradiance of 25–30 µmol photons m^2^ s^−1^ and long day photo-period (16:8 h light-dark conditions). The biomass was dried at 25 °C up to a 12% moisture content.

### 3.3. Total Lipid Extraction

Total lipid extraction was performed by using a modified Bligh and Dyer protocol [51,77]. A mixture of methanol/chloroform (2:1, per volume) was added to 250 mg of seaweed (three replicates). The mixture was transferred to a glass tube with a Teflon-lined screw cap and, after addition of 3.75 mL of a mixture methanol/chloroform (2:1, per volume), was homogenized and incubated on ice on a rocking platform shaker (Stuart equipment STR6, Bibby Scientific, Stone, UK) for 2 h and 30 min. The mixture was centrifuged (Selecta JP Mixtasel, Abrera, Barcelona, Spain) at 626× *g* for 10 min and the organic phase was collected. The biomass residue was re-extracted twice with 1.50 mL of a mixture methanol/chloroform (2:1, per volume). Water was added to the total collected organic phase, followed by centrifugation at 626× *g* for 10 min, and the organic (lower) phase was recovered. Solvents were dried under a stream of nitrogen gas. The total lipid extract content was estimated by gravimetry. Lipid extracts were stored at −20 °C prior to analysis by LC–MS and GC–MS.

### 3.4. Analysis of Fatty Acid Methyl Esters—FAMEs by Gas Chromatography–Mass Spectrometry (GC–MS)

Fatty acid methyl esters (FAMEs) were prepared from the total lipid extracts using a methanolic solution of potassium hydroxide (2.0 M) according to the methodology previously described [78]. 2.0 μL of a solution of hexane containing the FAMEs and 0.42 μg mL^−1^ of methyl heptadecanoate (Sigma, St. Louis, MO, USA) as internal standard were analyzed by gas chromatography-mass spectrometry (GC–MS). GC–MS data was acquired using an Agilent Technologies 6890 N Network (Santa Clara, CA, USA) equipped with a DB–FFAP column with the following specifications: 30 m long, 0.32 mm internal diameter, and 0.25 μm film thickness (123-3232, J&W Scientific, Folsom, CA, USA). The GC equipment was connected to an Agilent 5973 Network Mass Selective Detector operating with an electron impact mode at 70 eV and scanning the range *m*/*z* 40–500 in a 1 s cycle in a full scan mode acquisition. The oven temperature was programmed as follows: (1) the initial temperature was set up to 80 °C for 3 min; (2) a linear increase to 160 °C at 25 °C min^−1^; (3) a linear increase at 2 °C min^−1^ to 210 °C; and (4) a linear increase at 30 °C min^−1^ to 250 °C followed by 10 min at this temperature. The injector and detector temperatures were 220 and 280 °C, respectively. Helium was used as carrier gas at a flow rate of 1.7 mL min^−1^. At least, three replicates were performed. The identification of each FA was performed by mass spectrum comparison with those in the Wiley 275 library and confirmed by its interpretation and comparison with the literature (AOCS Lipid Library). Quantitative analysis of fatty acids was achieved from calibration curves of each methyl ester of fatty acids from a FAME mixture (Supelco 37 Component FAME Mix, CRM47885, Sigma Aldrich, St. Louis, MO, USA), analyzed by GC-MS under the same conditions of extracts and results expressed as mg kg^−1^ of dry biomass. The relative amounts of FAs were calculated by the ratio of the amount of each FAME and the sum of all identified FAMEs and results were expressed as means (%)*.*

### 3.5. Analysis of Polar Lipids by Hydrophilic Interaction Liquid Chromatography–Mass Spectrometry Based (LC–MS)

High performance LC (HPLC) system (Accela^TM^ Thermo Fisher Scientific, Waltham, MA, USA, country) with an autosampler coupled online to the Q-Exactive^®^ mass spectrometer with Orbitrap^®^ technology was used. The solvent system consisted of two mobile phases as follows: mobile phase A (acetonitrile:methanol:water 50:25:25 (per volume) with 1 mM ammonium acetate) and mobile phase B (acetonitrile:methanol 60:40 (per volume) with 1 mM ammonium acetate). Initially, 0% of mobile phase A was held isocratically for 8 min, followed by a linear increase to 60% of A within 7 min and a maintenance period of 15 min, returning to the initial conditions in 10 min. A volume of 5 µL of each sample containing 5 µg of lipid extract and 95 µL of eluent B was introduced into the Ascentis^®^ Si column (15 cm × 1 mm, 3 µm, Sigma-Aldrich) with a flow rate of 40 µL min^−1^ and at 30 °C. 

The mass spectrometer with Orbitrap^®^ technology was operated simultaneously in positive (electrospray voltage 3.0 kV) and negative (electrospray voltage −2.7 kV) modes with high resolution with 70,000 and automatic gain control (AGC) target of 1 × 10^6^, the capillary temperature was 250 °C and the sheath gas flow was 15 U. In MS/MS experiments, a resolution of 17,500 and AGC target of 1 × 10^5^ were used and the cycles consisted in one full scan mass spectrum and ten data-dependent MS/MS scans and were repeated continuously throughout the experiments with the dynamic exclusion of 60 s and intensity threshold of 1 × 10^4^. Normalized collision energy™ (CE) ranged between 25, 30 and 35 eV. Data acquisition was carried out using the Xcalibur data system (V3.3, Thermo Fisher Scientific, Waltham, MA, USA). At least, three replicates were performed. The identification of molecular species of polar lipids was based on the assignment of the molecular ions observed in LC–MS spectra and by the identification of the fragmentation pattern of each class observed in the MS/MS spectra of each ion [50,51,52]. To confirm the identification of molecular species, mass accuracy (Qual Browser) was determined with ≤5 ppm to confirm the elemental composition calculation of empirical formula (Supplementary Materials).

### 3.6. Nutritional Values

The nutritional quality of lipids was assessed by considering atherogenic and thrombogenic indexes, IA and IT respectively, calculated using the method described by Ulbricht and Southgate [46]:

IA = (12:0 + 4 × 14:0 + 16:0)/(Σ MUFA + Σ *n*-6 PUFA + Σ *n*-3 PUFA) (1)

IT = (14:0 + 16:0 + 18:0)/(0.5 × Σ MUFA + 0.5 × Σ *n*-6 PUFA + 3 × Σ *n*-3 PUFA + Σ *n*-3/*n*-6) (2)

## 4. Conclusions

This work reports for the first time the full characterization of the lipidome of *Porphyra dioica* blade and conchocelis life stages. Conchocelis had the highest total lipid content. By using high resolution liquid chromatography coupled to Q–Exactive mass spectrometry instrument, 110 and 100 lipid molecular species were identified in the lipidome of the blade and conchocelis, respectively, distributed by glycolipids, phospholipids, inositephosphoceramides, and betaine lipids. Our results showed that life cycle stages were characterized by a similar profile of glycolipids and that the distinct phenotypes were associated with different phospholipid (PC, PA, PE, and PG) and betaine lipids (DGTS) profiles. Differences were mainly due to the distinct number and abundances of molecular species suggesting that metabolic differences between the two life stages were more related to lipid shifts of extraplastidial origin than plastidial cell membranes. Our findings demonstrate that glycolipids mainly contribute with 20:4(*n*-6) and 20:5(*n*-3), some of which are recognized as bioactive molecules. Phospholipids are a source of *n*-3 and *n*-6 FA, namely octapolyenoic C_18_-series and precursors. Important classes such as PC, PG and PA contribute to the eicosapolyenoic acids pool, namely 20:4(*n*-6) and 20:5(*n*-3). *Porphyra dioica* have low number of molecular species of betaine lipids, contrary to other Rhodophyta such as the order of Gracilariales.

Both life stages displayed similar FA compositions, although blades contained higher amounts of SFA and PUFA, such as eicosapentaenoic acid 20:5(*n*-3), while conchocelis exhibited higher amounts of MUFA and PUFA such as 20:4(*n*-6) and 20:5(*n*-3). The *n*-6/*n*-3 PUFA ratio of both life stages was less than 2, in accordance with the recommended threshold (<4) suitable for consumption in human diet to prevent chronic diseases. The nutritional quality of *Porphyra dioica*, evaluated through atherogenicity and thrombogenicity indexes, was higher in blades (0.85 and 0.49, respectively) than in conchocelis (0.34 and 0.30, respectively). These features highlight the nutritional value and the potential of *Porphyra dioica* as a source of *n*-6 and *n*-3 FA. Also, the lipidome of both blades (gametophyte) and conchocelis (sporophyte) can be an important source of valuable lipids (e.g., glycolipids and phospholipids) for biotechnological applications. 

## Figures and Tables

**Table 1:** Fatty acids profile of lipid extract from blade and conchocelis stages of *Porphyra dioica*.

	Blade		Conchocelis	
Fatty Acids	Mean (mg kg^−1^ Dry Biomass) ± SD	(Mean %)	Mean (mg kg^−1^ Dry Biomass) ± SD	(Mean %)
14:0	12.32 ± 0.00	(0.60)	28.84 ± 0.83	(0.81)
15:0	20.04 ± 0.34	(0.98)	35.24 ± 0.13	(0.98)
16:0	882.1 ± 12.4	(43.2)	784.7 ± 21.1	(21.9)
18:0	27.85 ± 0.26	(1.36)	52.26 ± 8.00	(1.46)
**Σ SFA**	**942.3**	**46.1**	**901.0**	**25.2**
16:1(*n*-7)	39.83 ± 0.42	(1.95)	210.3 ± 5.06	(5.88)
18:1(*n*-9)	184.8 ± 17.7	(9.04)	366.1 ± 50.0	(10.2)
18:1(*n*-7)	44.27 ± 1.33	(2.17)	287.2 ± 22.7	(8.03)
20:1(*n*-9)	51.63 ± 1.33	(2.53)	76.75 ± 8.76	(2.14)
22:1(*n*-9)	4.972 ± 0.04	(0.24)	7.953 ± 1.26	(0.22)
**Σ MUFA**	**325.5**	**15.9**	**948.3**	**26.5**
18:2(*n*-6)	81.89 ± 4.06	(4.01)	171.6 ± 18.6	(4.80)
18:3(*n*-3)			16.62 ± 0.05	(0.46)
20:2(*n*-6)	18.44 ± 0.98	(0.90)	30.56 ± 2.70	(0.85)
20:3(*n*-6)	44.82 ± 2.27	(2.19)	179.8 ± 17.8	(5.02)
20:4(*n*-6)	112.4 ± 4.22	(5.50)	751.0 ± 87.3	(21.0)
20:5(*n*-3)	518.3 ± 14.2	(25.4)	579.5 ± 96.4	(16.2)
**Σ PUFA**	**775.9**	**38.0**	**1729**	**48.3**
Total	2044		3578	
*n*-6/*n*-3	0.50		1.90	
IA *	0.85		0.34	
IT *	0.49		0.30	

* IA-Index of atherogenicity; IT-Index of thrombogenicity.

**Table 2:** Identification of glycolipids in the lipidome of blade and conchocelis stages of *Porphyra dioica* by HILIC–LC–MS, mass accuracy and MS/MS. Monogalactosyl diacylglycerol (MGDG) and digalactosyl diacylglycerol (DGDG) molecular species were identified as [M + NH_4_]^+^ ions, and sulfoquinovosyl diacylglycerol (SQDG) and sulfoquinovosyl monoacylglycerol (SQMG) molecular species were identified as [M − H]^−^ ions.

**Galactolipids [M + NH_4_]^+^**	**Fatty Acyl Composition**		
***m*/*z _Theoretical_***	**(C:N)**	**Blade**	**Conchocelis**
772.594	MGDG (34:2)	18:2/16:0	18:2/16:0
774.609	MGDG (34:1)	18:1/16:0	**18:1/16:0**
794.578	MGDG (36:5)	**20:5/16:0**	**20:5/16:0**
796.594	MGDG (36:4)	20:4/16:0	**20:4/16:0**
800.624	MGDG (36:2)	18:2/18:0	18:2/18:0
802.641	MGDG (36:1)	-----------	18:1/18:0
934.647	DGDG (34:2)	18:2/16:0	18:2/16:0
936.662	DGDG (34:1)	18:1/16:0	**18:1/16:0**
956.631	DGDG (36:5)	**20:5/16:0**	**20:5/16:0**
972.626	DGDG (36:5-OH)	20:5-OH/16:0	20:5-OH/16:0
**Sulfolipids [M − H]^−^**	**Fatty Acyl Composition**		
***m*/*z _Theoretical_***	**(C:N)**	**Blade**	**Conchocelis**
555.284	SQMG 16:0	16:0	16:0
793.514	SQDG (32:0)	16:0/16:0	16:0/16:0
817.514	SQDG (34:2)	18:2/16:0	18:2/16:0
839.498	SQDG (36:5)	**20:5/16:0**	**20:5/16:0**
855.493	SQDG (36:5-OH)	20:5-OH/16:0	20:5-OH/16:0

Numbers in parentheses (C:N) indicate the number of carbon atoms (C) and number of double bonds (N) in the fatty acid side chains. Bold represents the abundant molecular species.

**Table 3:** Identification of phospholipids in the lipidome of blade and conchocelis stages of *Porphyra dioica* by HILIC–LC–MS, mass accuracy and MS/MS. Phosphatidylcholine (PC) and lyso-PC (LPC) molecular species were identified as [M + H]^+^ ions; phosphatidylethanolamine (PE), lyso-PE (LPE), phosphatidylglycerol (PG), lyso-PG (LPG), phosphatidic acid (PA), phosphatidylinositol (PI), and inositolphosphoceramide (IPC) molecular species were identified as [M − H]^−^ ions.

[M + H]^+^		Fatty Acyl Composition	
*m*/*z* *_Theoretical_*	(C:N)	Blade	Conchocelis
Phosphatidylcholine (PC)			
732.554	PC (32:1)	16:0/16:1 and 14:0/18:1	-----------
734.569	PC (32:0)	16:0/16:0 and 14:0/18:0	16:0/16:0
752.523	PC (34:5)	-----------	14:0/20:5
754.538	PC (34:4)	14:0/20:4 and 16:2/18:2	14:0/20:4 and 16:2/18:2
756.554	PC (34:3)	16:0/18:3 and 14:0/20:3	16:1/18:2
758.569	PC (34:2)	16:0/18:2 and 16:1/18:1	16:0/18:2 and 16:1/18:1
760.585	PC (34:1)	16:0/18:1	16:0/18:1
762.601	PC (34:0)	16:0/18:0	-----------
780.554	PC (36:5)	16:0/20:5 and 16:1/20:4	16:0/20:5
782.569	PC (36:4)	**16:0/20:4**	16:0/20:4
784.585	PC (36:3)	**16:0/20:3 and 18:1/18:2**	16:0/20:3 and 18:1/18:2
786.601	PC (36:2)	**18:1/18:1 and 18:0/18:2**	**18:1/18:1 and 18:0/18:2**
788.616	PC (36:1)	18:0/18:1	-----------
804.554	PC (38:7)	18:3/20:4 and 18:2/20:5	-----------
806.569	PC (38:6)	18:2/20:4 and 18:1/20:5	18:1/20:5
808.585	PC (38:5)	18:1/20:4	**18:1/20:4**
810.601	PC (38:4)	18:1/20:3	18:1/20:3
812.616	PC (38:3)	18:0/20:3	-----------
Lyso-phosphatidylcholine (LPC)			
494.339	LPC (16:1)	LPC (16:1)	------------_
496.323	LPC (16:0)	**LPC (16:0)**	**LPC (16:0)**
518.321	LPC (18:3)	LPC (18:3)	------------
522.355	LPC (18:1)	LPC (18:1)	LPC (18:1)
524.370	LPC (18:0)	LPC (18:0)	------------
Phosphatidylethanolamine (PE)			
660.460	PE (30:1)	14:0/16:1	14:0/16:1
662.477	PE (30:0)	14:0/16:0	14:0/16:0
686.477	PE (32:2)	16:1/16:1	16:1/16:1
688.492	PE (32:1)	**16:0/16:1**	**16:0/16:1**
712.492	PE (34:3)	16:0/18:3 and 16:1/18:2	16:0/18:3 and 16:1/18:2
714.508	PE (34:2)	16:1/18:1	**16:1/18:1**
716.524	PE (34:1)	16:0/18:1	16:0/18:1
736.492	PE (36:5)	16:0/20:5	16:0/20:5
738.508	PE (36:4)	**16:0/20:4**	-----------
740.524	PE (36:3)	-----------	18:0/18:3
742.539	PE (36:2)	-----------	18:1/18:1 and 18:0/18:2
744.555	PE (36:1)	-----------	18:0/18:1
786.508	PE (40:8)	20:4/20:4	-----------
Lyso-phosphatidylethanolamine (LPE)			
450.263	LPE (16:1)	LPE (16:1)	**LPE (16:1)**
452.278	LPE (16:0)	**LPE (16:0)**	LPE (16:0)
478.294	LPE (18:1)	LPE (18:1)	**LPE (18:1)**
Phosphatidylglycerol (PG)			
691.456	PG (30:1)	-----------	14:0/16:1
717.471	PG (32:2)	-----------	16:0/16:2 and 16:1/16:1
719.487	PG (32:1)	16:1/16:0 and (14:0/18:1)	**16:1/16:0 and 14:0/18:1**
721.502	PG (32:0)	16:0/16:0 and 14:0/18:0	16:0/16:0 and 14:0/18:0
745.503	PG (34:2)	-----------	**16:1/18:1 and 14:0/18:2**
747.518	PG (34:1)	16:0/18:1 and 18:0/16:1	16:0/18:1 and 18:0/16:1
749.534	PG (34:0)	-----------	16:0/18:0
765.471	PG (36:6)	16:1/20:5 and 18:3/18:3	16:1/20:5 and 18:3/18:3
767.487	PG (36:5)	**16:0/20:5 and 16:1/20:4**	16:0/20:5 and 16:1/20:4
769.503	PG (36:4)	**16:0/20:4 and 18:1/18:3 and 18:2/18:2**	16:0/20:4 and 18:1/18:3 and 18:2/18:2
771.518	PG (36:3)	18:1/18:2 and 16:0/20:3	18:1/18:2 and 16:0/20:3
773.534	PG (36:2)	18:1/18:1 and 16:0/20:2	**18:1/18:1 and 16:0/20:2**
775.549	PG (36:1)	**18:0/18:1**	**18:0/18:1 and 16:0/20:1**
801.565	PG (38:2)	16:0/22:2	16:0/22:2
803.580	PG (38:1)	16:0/22:1	16:0/22:1
Lyso-phosphatidylglycerol (LPG)			
481.257	LPG (16:1)	LPG (16:1)	LPG (16:1)
483.273	LPG (16:0)	**LPG (16:0)**	**LPG (16:0)**
509.289	LPG (18:1)	LPG (18:1)	**LPG (18:1)**
Phosphatidic acid (PA)			
643.434	PA (32:2))	14:0/18:2 and 16:1/16:1	-----------
645.450	PA (32:1)	14:0/18:1 and 16:0/16:1	14:0/18:1 and 16:0/16:1
647.466	PA (32:1)	16:0/16:0	-----------
669.450	PA (34:3)	16:0/18:3 and 16:1/18:2 14:0/20:3	-----------
671.465	PA (34:2)	16:0/18:2	**16:0/18:2**
673.481	PA (34:1)	16:1/18:0 and 16:0/18:1	-----------
691.434	PA (36:6)	16:1/20:5	-----------
693.450	PA (36:5)	16:0/20:5	-----------
695.466	PA (36:4)	16:0/20:4	16:0/20:4
697.481	PA (36:3)	16:0/20:3	-----------
699.497	PA (36:2)	16:0/20:2	16:0/20:2
701.513	PA (36:1)	**16:0/20:1**	16:0/20:1
Phosphatidylinositol (PI)			
831.503	PI (34:3)	-----------	16:0/18:3
833.519	PI (34:2)	**16:0/18:2 and 16:1/18:1**	**16:0/18:2 and 16:1/18:1**
835.534	PI (34:1)	16:0/18:1	16:0/18:1
883.534	PI (38:5)	-----------	18:0/20:5
Inositephosphoceramide (IPC)			
918.681	IPC (d44:1)	d18:1/26:0	-----------
920.623	IPC (t42:2-OH)	**t18:1/24:1-OH**	t18:1/24:1-OH
922.639	IPC (t42:1-OH)	t18:0/24:1-OH	t18:0/24:1-OH
924.654	IPC (t42:0-OH)	t18:0/24:0-OH	t18:0/24:0-OH

Numbers in parentheses (C:N) indicate the number of carbon atoms (C) and number of double bonds (N) in the fatty acid side chains. Bold represents the abundant molecular species.

**Table 4:** Identification of betaine lipids in the lipidome of blade and conchocelis stages of *Porphyra dioica* by HILIC–LC–MS, mass accuracy and MS/MS. Diacyglyceryl-*N*,*N*,*N*-trimethyl homoserine (DGTS) molecular species were identified as [M + H]^+^ ions.

[M + H]^+^	Fatty Acyl Composition		
*m*/*z _Theoretical_*	(C:N)	Blade	Conchocelis
682.562	DGTS (30:1)	-----------	14:0/16:1
684.578	DGTS (30:0)	-----------	14:0/16:0
710.593	DGTS (32:1)	**16:0/16:1 and 14:0/18:1**	**16:0/16:1 and 14:0/18:1**
736.609	DGTS (34:2)	16:0/18:2	16:0/18:2
738.625	DGTS (34:1)	-----------	16:0/18:1

Numbers in parentheses (C:N) indicate the number of carbon atoms (C) and number double bonds (N) in the fatty acid side chains. Bold represents the abundant molecular species.

## Supplementary Materials

The Supplementary Materials are available online.

## References

[1] Sahoo S., Tang X., Yarish C. (2002). *Porphyra*—The economic seaweed as a new experimental system. Curr. Sci..

[2] Blouin N.A., Brodie J.A., Grossman A.C., Xu P., Brawley S.H. (2011). *Porphyra*: A marine crop shaped by stress. Trends Plant Sci..

[3] WoRMS Editorial Board (2017). World Register of Marine Species. http://www.marinespecies.org.

[4] Pereira R., Kraemer G., Yarish C., Sousa-Pinto I. (2008). Nitrogen uptae by gametophytes of *Porphyra dioica* (Bangiales, Rhodophyta) under controlled-culture conditions. Eur. J. Phycol..

[5] Pereira R., Sousa-Pinto I., Yarish C. (2004). Field and culture studies of the life history of *Porphyra dioica* (Bangiales, Rhodophyta) from Portugal. Phycologia.

[6] Drew K.M. (1954). Studies in the Bangioideae. Ann. Bot..

[7] Candia A., Lindstrom S., Reyes E. (1999). *Porphyra* sp. (Bangiales, Rhodophyta): Reproduction and life form. Hydrobiologia.

[8] Stengel D.B., Connan S., Popper Z.A. (2011). Algal chemodiversity and bioactivity: Sources of natural variability and implications for commercial application. Biotechnol. Adv..

[9] Stabili L., Acquaviva M.I., Biandolino F., Cavallo R.A., De Pascali S.A., Fanizzi F.P., Narracci M., Petrocelli A., Cecere E. (2012). The lipidic extract of the seaweed *Gracilariopsis longissima* (Rhodophyta, Gracilariales): A potential resource for biotechnological purposes?. New Biotechnol..

[10] Zhang W., Gao J.T., Zhang Y.C., Qin S. (2006). Optimization of conditions for cell cultivation of *Porphyra haitanensis* conchocelis in a bubble-column bioreactor. World J. Microbiol. Biotechnol..

[11] Van Ginneken V.J.T., Helsper J.P.F.G., de Visser W., Van Keulen H., Brandenburg W.A. (2011). Polyunsaturated fatty acids in various macroalgal species from North Atlantic and tropical seas. Lipids Health Dis..

[12] Harwood J.L., Guschina I.A. (2009). The versatility of algae and their lipid metabolism. Biochimie.

[13] Plouguerné E., da Gama B.A.P., Pereira R.C., Barreto-Bergter E. (2014). Glycolipids from seaweeds and their potential biotechnological applications. Front. Cell. Infect. Microbiol..

[14] Banskota A.H.A.H., Stefanova R., Sperker S., Lall S., Craigie J.S.J.S., Hafting J.T.J.T. (2014). Lipids isolated from the cultivated red alga *Chondrus crispus* inhibit nitric oxide production. J. Appl. Phycol..

[15] Banskota A.H., Stefanova R., Sperker S., Lall S.P., Craigie J.S., Hafting J.T., Critchley A.T. (2014). Polar lipids from the marine macroalga *Palmaria palmata* inhibit lipopolysaccharide-induced nitric oxide production in RAW264.7 macrophage cells. Phytochemistry.

[16] Robertson R.C., Guihéneuf F., Bahar B., Schmid M., Stengel D.B., Fitzgerald G.F., Ross R.P., Stanton C. (2015). The anti-Inflammatory effect of algae-derived lipid extracts on lipopolysaccharide (LPS)-stimulated human THP-1 macrophages. Mar. Drugs.

[17] Eitsuka T., Nakagawa K., Igarashi M., Miyazawa T. (2004). Telomerase inhibition by sulfoquinovosyldiacylglycerol from edible purple laver (*Porphyra yezoensis*). Cancer Lett..

[18] Chen C.Y., Chou H.N. (2002). Screening of red algae filaments as a potential alternative source of eicosapentaenoic acid. Mar. Biotechnol..

[19] Xiaolei F., Guangce W., Demao L., Pu X., Songdong S. (2008). Study on early-stage development of conchospore in *Porphyra yezoensis* Ueda. Aquaculture.

[20] Wang X., Zhao P., Luo Q., Yan X., Xu J., Chen J., Chen H. (2015). Metabolite changes during the life history of *Porphyra haitanensis*. Plant Biol..

[21] Spolaore P., Joannis-Cassan C., Duran E., Isambert A. (2006). Commercial applications of microalgae. J. Biosci. Bioeng..

[22] Soler-Vila A., Coughlan S., Guiry M.D., Kraan S. (2009). The red alga *Porphyra dioica* as a fish-feed ingredient for rainbow trout (*Oncorhynchus mykiss*): Effects on growth, feed efficiency, and carcass composition. J. Appl. Phycol..

[23] Rulong L. (2015). *Pyropia* conchocelis: Potential as an algal source for carotenoid extraction. Am. J. BioSci..

[24] Leal M.C., Munro M.H.G., Blunt J.W., Puga J., Jesus B., Calado R., Rosa R., Madeira C. (2013). Biogeography and biodiscovery hotspots of macroalgal marine natural products. Nat. Prod. Rep..

[25] Bhakuni D.S., Rawat D.S. (2005). Bioactive metabolites of marine algae, fungi and bacteria. Bioactive Marine Natural Products.

[26] Wu D., Fujio M., Wong C.H. (2008). Glycolipids as immunostimulating agents. Bioorgan. Med. Chem..

[27] Burri L., Hoem N., Banni S., Berge K. (2012). Marine omega-3 phospholipids: Metabolism and biological activities. Int. J. Mol. Sci..

[28] Calder P.C. (2014). Marine omega-3 fatty acids and inflammatory processes: Effects, mechanisms and clinical relevance. Biochim. Biophys. Acta.

[29] El Baky H.H.A., El Baz F.K., El Baroty G.S., Asker M.M.S., Ibrahim E.A. (2014). Phospholipids of some marine microalgae: Identification, antivirus, anticancer and antimicrobial bioactivities. Der Pharma Chem..

[30] Lu F.S.H., Nielsen N.S., Baron C.P., Jacobsen C. (2017). Marine phospholipids: The current understanding of their oxidation mechanisms and potential uses for food fortification. Crit. Rev. Food Sci. Nutr..

[31] Cian R.E., Fajardo M.A., Alaiz M., Vioque J., González R.J., Drago S.R. (2014). Chemical composition, nutritional and antioxidant properties of the red edible seaweed *Porphyra columbina*. Int. J. Food Sci. Nutr..

[32] Sánchez-Machado D.I., López-Cervantes J., López-Hernández J., Paseiro-Losada P. (2004). Fatty acids, total lipid, protein and ash contents of processed edible seaweeds. Food Chem..

[33] Schmid M., Guihéneuf F., Stengel D.B. (2014). Fatty acid contents and profiles of 16 macroalgae collected from the Irish Coast at two seasons. J. Appl. Phycol..

[34] Fleurence J., Gutbier G., Mabeau S., Leray C. (1994). Fatty acids from 11 marine macroalgae of the French Brittany coast. J. Appl. Phycol..

[35] Luo Q., Zhu Z., Zhu Z., Yang R., Qian F., Chen H., Yan X. (2014). Different responses to heat shock stress revealed heteromorphic adaptation strategy of *Pyropia haitanensis* (Bangiales, Rhodophyta). PLoS ONE.

[36] Simopoulos A. (2002). The importance of the ratio of omega-6/omega-3 essential fatty acids. Biomed. Pharmacother..

[37] Kumari P., Reddy C.R.K., Jha B. (2011). Comparative evaluation and selection of a method for lipid and fatty acid extraction from macroalgae. Anal. Biochem..

[38] Calder P.C., Albers R., Antoine J.-M., Blum S., Ferns G.A., Folkerts G., Bourdet-Sicard R., Friedmann P.S., Frost G.S., Guarner F. (2009). Inflammatory disease processes and interactions with nutrition. Br. J. Nutr..

[39] Calder P.C. (2013). Omega-3 polyunsaturated fatty acids and inflammatory processes: Nutrition or pharmacology?. Br. J. Clin. Pharmacol..

[40] De Roos B., Mavrommatis Y., Brouwer I.A. (2009). Long-chain *n*-3 polyunsaturated fatty acids: New insights into mechanisms relating to inflammation and coronary heart disease. Br. J. Pharmacol..

[41] Lordan S., Ross R.P., Stanton C. (2011). Marine bioactives as functional food ingredients: Potential to reduce the incidence of chronic diseases. Mar. Drugs.

[42] Calder P.C. (2017). Omega-3 fatty acids and inflammatory processes: From molecules to man. Biochem. Soc. Trans..

[43] Pereira H., Barreira L., Figueiredo F., Custódio L., Vizetto-Duarte C., Polo C., Rešek E., Engelen A., Varela J. (2012). Polyunsaturated Fatty acids of marine macroalgae: Potential for nutritional and pharmaceutical applications. Mar. Drugs.

[44] Simopoulos A.P. (2008). The importance of the omega-6/omega-3 fatty acid ratio in cardiovascular disease and other chronic diseases. Exp. Biol. Med..

[45] Simopoulos A.P. (1999). Essential fatty acids in health and chronic disease. Am. J. Clin. Nutr..

[46] Ulbricht T.L.V., Southgate D.A.T. (1991). Coronary heart disease: Seven dietary factors. Lancet.

[47] Kumar M., Kumari P., Trivedi N., Shukla M.K., Gupta V., Reddy C.R.K., Jha B. (2010). Minerals, PUFAs and antioxidant properties of some tropical seaweeds from Saurashtra coast of India. J. Appl. Phycol..

[48] Guschina I.A., Harwood J.L. (2006). Lipids and lipid metabolism in eukaryotic algae. Prog. Lipid Res..

[49] Khotimchenko S.V. (2002). Distribution of glyceroglycolipids in marine algae and grasses. Chem. Nat. Compd..

[50] Melo T., Alves E., Azevedo V., Martins A.S., Neves B., Domingues P., Calado R., Abreu M.H., Domingues M.R. (2015). Lipidomics as a new approach for the bioprospecting of marine macroalgae—Unraveling the polar lipid and fatty acid composition of *Chondrus crispus*. Algal Res..

[51] Da Costa E., Melo T., Moreira A., Bernardo C., Helguero L., Ferreira I., Cruz M., Rego A., Domingues P., Calado R. (2017). Valorization of lipids from *Gracilaria* sp. through lipidomics and decoding of antiproliferative and anti-Inflammatory activity. Mar. Drugs.

[52] Da Costa E., Melo T., Moreira A.S.P., Alves E., Domingues P., Calado R., Abreu M.H.M.H., Domingues M.R. (2015). Decoding bioactive polar lipid profile of the macroalgae *Codium tomentosum* from a sustainable IMTA system using a lipidomic approach. Algal Res..

[53] Kumari P., Reddy R., Jha B. (2014). Quantification of selected endogenous hydroxy-oxylipins from tropical marine macroalgae. Mar. Biotechnol..

[54] Khozin-Goldberg I., Cohen Z. (2011). Unraveling algal lipid metabolism: Recent advances in gene identification. Biochimie.

[55] Wang Z., Benning C. (2012). Chloroplast lipid synthesis and lipid trafficking through ER-plastid membrane contact sites. Biochem. Soc. Trans..

[56] Tsai C., Pan B.S. (2012). Identification of sulfoglycolipid bioactivities and characteristic fatty acids of marine macroalgae. JAFC.

[57] Mühlroth A., Li K., Røkke G., Winge P., Olsen Y., Hohmann-Marriott M.F., Vadstein O., Bones A.M. (2013). Pathways of lipid metabolism in marine algae, co-expression network, bottlenecks and candidate genes for enhanced production of EPA and DHA in species of Chromista. Mar. Drugs.

[58] Wang X., Su X., Luo Q., Xu J., Chen J., Yan X., Chen H. (2014). Profiles of glycerolipids in *Pyropia haitanensis* and their changes responding to agaro-oligosaccharides. J. Appl. Phycol..

[59] Birner R., Bürgermeister M., Schneiter R., Daum G. (2001). Roles of phosphatidylethanolamine and of its several biosynthetic pathways in *Saccharomyces cerevisiae*. Mol. Biol. Cell.

[60] Thompson G.A. (1996). Lipids and membrane function in green algae. Biochim. Biophys. Acta Lipids Lipid Metab..

[61] Gerasimenko N.I., Busarova N.G., Moiseenko O.P. (2010). Age-dependent changes in the content of lipids, fatty acids, and pigments in brown alga *Costaria costata*. Russ. J. Plant Physiol..

[62] Munnik T. (2001). Phosphatidic acid: An emerging plant lipid second messenger. Trends Plant Sci..

[63] Testerink C., Munnik T. (2005). Phosphatidic acid: A multifunctional stress signaling lipid in plants. Trends Plant Sci..

[64] Dembitsky V.M., Rozentsvet O.A. (1990). Phospholipid composition of some marine red algae. Phytochemistry.

[65] Mueller-Roeber B. (2002). Inositol Phospholipid Metabolism in *Arabidopsis*. Characterized and putative isoforms of inositol phospholipid kinase and phosphoinositide-specific phospholipase C. Plant Physiol..

[66] Munnik T., Nielsen E. (2011). Green light for polyphosphoinositide signals in plants. Curr. Opin. Plant Biol..

[67] Michell R.H. (2011). Inositol and its derivatives: Their evolution and functions. Adv. Enzyme Regul..

[68] Khotimchenko S.V. (2005). Lipids from the marine alga *Gracilaria verrucosa*. Chem. Nat. Compd..

[69] Khotimchenko S.V. (2006). Variations in lipid composition among different developmental stages of *Gracilaria verrucosa* (Rhodophyta). Bot. Mar..

[70] Paul-André S., Norio M., Joyard J., Maréchal E., Miège C., Block M., Dorne A.-J., Douce R. (2004). Structure, Distribution and biosynthesis of glycerolipids from higher plant chloroplasts. Lipids Photosynth. Struct. Funct. Genet..

[71] Levchenko E.V. (2003). Carbon metabolism transitions during the development of marine macroalga *Gracilaria verrucosa*. Russ. J. Plant Physiol..

[72] Kunzler K., Eichenberger W. (1997). Betaine lipids and zwitterionic phospholipids in plants and fungi. Phytochemistry.

[73] Sato N. (1992). Betaine Lipids. Bot. Mag. Tokyo.

[74] Kato M., Sakai M., Adachi K., Ikemoto H., Sano H. (1996). Distribution of betaine lipids in marine algae. Phytochemistry.

[75] Dembitsky V.M. (1996). Betaine ether-linked glycerolipids: Chemistry and biology. Prog. Lipid Res..

[76] Li S., Xu J., Chen J.J., Chen J.J., Zhou C., Yan X. (2014). The major lipid changes of some important diet microalgae during the entire growth phase. Aquaculture.

[77] Bligh E.G., Dyer W.J. (1959). A rappid method of total lipid extraction and purification. Can. J. Biochem. Physiol..

[78] Aued-Pimentel S., Lago J.H.G., Chaves M.H., Kumagai E.E. (2004). Evaluation of a methylation procedure to determine cyclopropenoids fatty acids from Sterculia striata St. Hil. Et Nauds seed oil. J. Chromatogr. A.

