# Supplementary material for: High-Resolution Lipidomics of the Early Life Stages of the Red Seaweed Porphyra dioica
Source: Molecules. 2018 Jan 17;23(1):187. doi: 10.3390/molecules23010187 (PMC6017436; doi:10.3390/molecules23010187)
Supplement: Supplementary file 1 [file molecules-23-00187-s001.pdf]

High-Resolution Lipidomics of the Early Life Stages of the Red Seaweed *Porphyra dioica*

Elisabete da Costa [1], Vitor Azevedo [1], Tânia Melo [1], Andreia M. Rego [2], Dmitry V. Evtuguin [3], Pedro Domingues [1], Ricardo Calado [4], Rui Pereira [2], Maria H. Abreu [2], Maria R. Domingues [1,*]

Supplementary Information

Table S1. Molecular species observed by HILIC–MS, with the assignment of the total fatty acyl composition of each lipid molecular species. Analyses were determined by mass accuracy (error ≤ 5 ppm) using the Xcalibur software, exact mass calculator http: //www.sisweb.com/referenc/tools/ exactmass.htm, and lipid maps tools http://www.lipidmaps.org/tools

Porphyra dioica		Mass error (ppm, ≤ 5 ppm)		
**Glycolipids**	***m/z* theoretical**	**Blade**	**Conchocelis**	**Identification**
**Sulfolipids [M - H]⁻**				
C25 H47 O11 S	555.2839	-0.7010	0.3110	SQMG 16:0
C41 H77 O12 S	793.5136	-1.0770	-0.3210	SQDG (32:0)
C43 H77 O12 S	817.5136	-1.1560	1.9510	SQDG (34:2)
C45 H75 O12 S	839.4979	-1.5180	-0.7920	SQDG (36:5)
C45H75O13S	855.4928	-1.5190	-0.2680	SQDG (36:5-OH)
**Galactolipids [M + NH_4]$^+$**				
C43H82O10N	772.5939	-3.2540	-4.6650	MGDG (34:2)
C43H84O10N	774.6095	-2.4190	-3.8910	MGDG (34:1)
C45H80NO10	794.5782	-4.0575	-4.4351	MGDG (36:5)
C45H82NO10	796.5939	-2.6410	-2.7920	MGDG (36:4)
C45H86NO10	800.6240	-4.9206	-4.8712	MGDG (36:2)
C45H88NO10	802.6408	-------------	-4.9140	MGDG (36:1)
C49 H92 O15 N	934.6467	-3.4210	-2.2650	DGDG (34:2)
C49 H94 O15 N	936.6618	-3.8435	-2.6660	DGDG (34:1)
C51 H90 O15 N	956.6310	-3.6054	-0.3648	DGDG (36:5)
C51H90O16 N	972.6260	-3.9728	-1.0939	DGDG (36:5-OH)
**Phospholipids**	***m/z theoretical***	**Blade**	**Conchocelis**	**Identification**
**Phosphatidylcholine [M + H]$^+$**				
C40H79O8NP	732.5538	-3.2762	------------	PC (32:1)
C42H73O8NP	734.5695	-1.6336	-2.5866	PC (32:0)
C42H75O8NP	752.5225	------------	-2.6577	PC (34:5)
C42H77O8NP	754.5382	-1.7229	-3.1808	PC (34:4)
C42H79O8NP	756.5538	-3.0401	-2.7758	PC (34:3)
C42H81O8NP	758.5695	-3.4275	-2.7684	PC (34:2)
C42H83O8NP	760.5851	-3.4184	-2.6296	PC (34:1)
C44H75O8NP	762.6008	-4.1962	------------	PC (34:0)
C44H79O8NP	780.5538	-3.7153	-4.0997	PC (36:5)

C44H81O8NP	782.5695	-3.7058	-3.9613	PC (36:4)
C44H83O8NP	784.5851	-3.5688	-2.6766	PC (36:3)
C44H85O8NP	786.6008	-3.5596	-3.3054	PC (36:2)
C44H87O8NP	788.6164	-4.8186	------------	PC (36:1)
C46H79O8NP	804.5538	-4.8474	------------	PC (38:7)
C46H81O8NP	806.5695	-4.9593	-4.4634	PC (38:6)
C46H83O8NP	808.5851	-4.9469	-4.4522	PC (38:5)
C46H85O8NP	810.6008	-2.4673	-4.9346	PC (38:4)
C46H87O8NP	812.6164	-4.4302	------------	PC (38:3)
**Lyso-Phosphatidylcholine [M + H]$^+$**				
C24H49NO7P	494.3241	-2.4276	------------	LPC(16:1)
C24H51NO7P	496.3398	-2.6192	-3.6266	LPC (16:0)
C26H49NO7P	518.3241	-4.6303	------------	LPC (18:3)
C26H53NO7P	522.3554	-2.1059	-0.5743	LPC (18:1)
C26H55NO7P	524.3711	-1.7163	------------	LPC (18:0)

Phospholipids	*m/z theoretical*	Blade	Conchocelis	Identification
**Lyso-Phosphatidylglycerol [M - H]$^-$**				
C22H42O9P	481.2572	-2.0779	-1.4545	LPG (16:1)
C22H44O9P	483.2728	-5.8179	-1.2467	LPG (16:0)
C24H46O9P	509.2885	-2.3562	-1.7672	LPG (18:1)
**Phosphatidylglycerol [M - H]$^-$**				
C36H68O10P	691.4556	------------	-0.0782	PG (30:1)
C38H70O10P	717.4712	------------	-2.0964	PG (32:2)
C38H72O10P	719.4869	-2.8551	-1.6042	PG (32:1)
C38H74O10P	721.5025	-2.6393	-2.6393	PG (32:0)
C40H74O10P	745.5025	-2.6884	-1.4812	PG (34:2)
C40H76O10P	747.5182	-2.3468	-2.3468	PG (34:1)
C40H78O10P	749.5338	-----------	-4.9422	PG (34:0)
C42H70O10P	765.4712	-2.8794	-1.9649	PG (36:6)
C42H72O10P	767.4869	-3.1977	-1.8947	PG (36:5)
C42H74O10P	769.5025	-4.2940	-2.8645	PG (36:4)
C42H76O10P	771.5182	-2.7923	-2.1442	PG (36:3)
C42H78O10P	773.5338	-2.5912	-2.0741	PG (36:2)
C42H80O10P	775.5495	-3.1648	-2.3911	PG (36:1)
C44H82O10P	801.5651	-4.1170	-0.9980	PG (38:2)
C44H84O10P	803.5802	-1.6178	-1.3689	PG (38:1)
**Phosphatidic acid [M - H]$^-$**				
C35H64O8P	643.4344	-4.2346	------------	PA (32:2)
C35H66O8P	645.4501	0.9687	-0.8904	PA (32:1)
C35H68O8P	647.4657	-0.9680	------------	PA (32:0)
C37H66O8P	669.4501	2.5771	------------	PA (34:3)
C37H68O8P	671.4652	-0.1191	-0.8638	PA (34:2)
C37H70O8P	673.4814	-1.4475	------------	PA (34:1)
C39H64O8P	691.4344	-1.9158	------------	PA (36:6)

C39H66O8P	693.4501	-2.1267	------------	PA (36:5)
C39H68O8P	695.4652	-1.1216	3.6235	PA (36:2)
C39H70O8P	697.4814	-1.4337	------------	PA (36:3)
C39H72O8P	699.4970	-1.5726	-2.1444	PA (36:2)
C39H74O8P	701.5127	-1.4255	-1.8531	PA (36:1)
**Phosphatidylethanolamine [M - H]$^-$**				
C35H67O8NP	660.4610	1.5538	-4.3512	PE (30:1)
C35H69O8NP	662.4766	2.9830	-3.8097	PE (30:0)
C37H69O8NP	686.4766	-1.4914	-0.9088	PE (32:2)
C37H71O8NP	688.4923	-1.4145	-1.8503	PE (32:1)
C39H71O8NP	712.4923	-2.0687	-2.7704	PE (34:3)
C39H73O8NP	714.5079	-0.5934	-1.5731	PE (34:2)
C39H75O8NP	716.5236	1.1527	4.9209	PE (34:1)
C41H71O8NP	736.4923	-1.0508	-1.7297	PE (36:5)
C41H73O8NP	738.5079	-0.9803	------------	PE (36:4)
C41H75O8NP	740.5236	------------	-2.1256	PE (36:3)
C41H77O8NP	742.5392	------------	-2.0525	PE (36:2)
C41H79O8NP	744.5549	------------	2.3179	PE (36:1)
C45H73O8NP	786.5079	2.7667	------------	PE (40:8)
**Lyso-Phosphatidylethanolamine [M - H]$^-$**				
C21H41NO7P	450.2626	-1.3326	0.8884	LPE (16:1)
C21H43NO7P	452.2782	-1.1055	-0.4422	LPE (16:0)
C23H45NO7P	478.2939	-1.6726	-1.8817	LPE (18:1)
**Phosphatidylinositol [M - H]$^-$**				
C43H76O13P	831.5029	------------	-4.2070	PI (34:3)
C43H78O13P	833.5185	-4.3769	-4.2569	PI (34:2)
C43H80O13P	835.5342	-2.1523	1.1989	PI (34:1)
C47H80O13P	883.5342	------------	-3.5067	PI (38:5)
**Inositephosphoceramide [M - H]$^-$**				
C50H97NO11P	918.6805	-2.3947	------------	IPC (d44:1)
C48H99NO11P	920.6228	-1.3111	-1.4197	IPC (t42:2-OH)
C48H93NO13P	922.6385	-3.8553	-4.1804	IPC (t42:1-OH)
C48H95NO13P	924.6541	0.7495	1.7228	IPC (t42:0-OH)

**Betaine lipids [M + H]$^+$**	***m/z* theoretical**	**Blade**	**Conchocelis**	**Identification**
C40H76O7N	682.5622	-------------	-2.606	DGTS (30:1)
C40H78O7N	684.5778	-------------	-4.571	DGTS (30:0)
C42H80NO7	710.5935	-4.755	-2.504	DGTS (32:1)
C44H82O7N	736.6091	-3.703	-3.568	DGTS (34:2)
C44H84O7N	738.6248	-------------	0.406	DGTS (34:1)
